# A Telechelic Fluorescent Indicator Based on Polymer Conformational Change for Free Copper(II) Ions

**DOI:** 10.3390/s23239476

**Published:** 2023-11-28

**Authors:** Yuan Chen, Bo Si, Noah Cote, Roy P. Planalp, Rudi Seitz

**Affiliations:** Department of Chemistry, University of New Hampshire, 23 Academy Way, Durham, NH 03824, USA; yuan.chen@unh.edu (Y.C.); bo.si@unh.edu (B.S.); noah.cote@unh.edu (N.C.); roy.planalp@unh.edu (R.P.P.)

**Keywords:** free copper ions, RAFT polymerization, FRET, polymer conformational change

## Abstract

A novel copper(II) ion indicator based on polymer conformational change is designed and its chemo-response to the target analyte is tested in this paper. The word ‘telechelic’ in the title means that a polymer has two different fluorophores on either end. If one of them is a fluorescent donor and the other is a fluorescent acceptor, then the extent of Foerster resonance energy transfer (FRET) will depend on polymer conformation. The sensitivity of these sensors is tunable based on the chain length and the amount of the receptor on the polymer. This is revealed by the fluorescence response of 30mer, 50mer, and 100mer of poly(N-isopropyl)acrylamide with different amounts of metal chelation monomers. We also address the change in fluorescence over time due to the untangling of poly(N-isopropylacrylamide) in water. The fluorescent signal can maintain stability after metal binding. The photoluminescence results agree with the length calculation of polyelectrolytes. A fluorescent standard curve is created for the measurement of different concentrations of copper ions. The sensing limit can reach 10^−10^ M analytes, which is suitable for the measurement of chemicals in trace amounts in the environment.

## 1. Introduction

The bioaccumulation of toxic metal ions is chronic, especially in animals at the top of the food chain. Bioavailable metal ions are the proportion of total metal ions that are available for incorporation into biota [1]. Bioavailable metal ions are toxic because they are unbonded ions or ions free of natural ligands [2]. This part of the metal ions is small in concentration compared with the natural abundance of the total metal ions. Because of this, total metal ion measurements are not an accurate measure of metal ion toxicity [3]. 

Among these metal ions, copper(II) is the most common ion in the environment, especially in seawater. Copper has redox properties and is a structural and catalytic component for the protein and enzyme molecules involved in metabolic processes [4]. The toxicity of copper resides in its ability to form free radicals, which cause oxidative stress [5].

A few methods have developed to measure bioavailable metal ions. A well-known biological method is a biotic ligand model [6,7]. It treats the bonding between the metal ions and the biological receptor as a metal–ligand system. From this test, the bioavailable copper(II) ions can range from 10^−5.5^ M to 10^−10^ M [8]. However, the measurement of free metal ions from this model is indirect and the response depends on many biological factors [9]. Therefore, the concentration of bioavailable copper ions tested by the biotic ligand model can only be taken as an approximate value for reference.

Direct methods like spectroscopic techniques [10] or voltammetry [11] are good methods to measure metal ions in trace concentrations. However, because these measurements respond to total metal rather than bioavailable metal ions, they are of limited value in assessing toxicity. Also, the equilibrium between free metal ions and natural metal ion complexes may be disturbed by the measurement, especially in the case of voltammetry. 

There are solution-based technologies that are suitable for the testing of free metal ions like ion-exchange membranes [12], diffusion gradient membranes [13], or resin [14]. However, these methods largely rely on separation techniques and sample loss in the separation step may reduce the detection accuracy. Furthermore, the concentration of metal to be measured is very low, resulting in errors due to contamination. Therefore, we require a more direct and convenient solution-based technique. A metal ion indicator is a good option. Like a pH indicator, the concentration of metal ions can easily be derived from a standard curve of a certain ion. The immediate color change of the indicator is also good for a quick screen of batch samples by a visual inspection. 

One of the issues that has to be addressed to measure uncomplexed metal ions is sensitivity because free concentrations are often nanomolar and below. Fluorescence is a method that has the required sensitivity. However, Cu(II) is a metal ion that is not amenable to fluorescent-sensing because it usually quenches emissions. Some researchers have utilized this intrinsic property of Cu(II) to design ‘turn-off’ sensors for Cu(II) [15]. However, there are a lot of pathways that lead to fluorescence-quenching, especially in a complex solution matrix. This introduces undetectable errors in the measurement. 

Our approach, based on the measurement of a Forster resonance energy transfer (FRET) signal, overcomes the quenching problem. FRET is an energy transfer pathway between two fluorophores [16,17,18]. If the emission wavelength from one fluorophore (donor) overlaps with the absorption of another (acceptor), the donor can transfer its energy to the acceptor. The transfer efficiency is directly related to the distance between these two fluorophores. The closer these two fluorophores, the higher the FRET efficiency. FRET has been widely used in the investigation of protein unfolding in biological studies [19]. The prospect of a sub-nanometer resolution provides a FRET microscope with the ability to map the kinetics and interactions of proteins [20,21].

We developed a method to sense Cu(II) ions by mixing two strands of fluorophore-labeled poly(N-isopropylacrylamide) (pNIPAM) [22,23]. One strand was labeled with the donor fluorophore and the other with the acceptor. Bipyridine monomers that can bind with Cu(II) were copolymerized with N-isopropylacrylamide. Poly(N-isopropylacrylamide) undergoes a thermal phase transition at elevated temperatures, which leads to aggregation and precipitation [24]. The temperature at which the phase transition occurs is defined as the lower critical solution temperature (LCST). Above pNIPAM’s LCST without Cu(II), pNIPAM strands associate and we observed FRET, which led to acceptor emissions even though we chose an excitation wavelength that excited the donor. Adding Cu(II) at a high enough concentration caused it to complex with bipyridine, introducing a positive charge into the polymer that caused the chains to separate. This led to much less FRET [22]. The problem was that if the polymer concentration was too low, then the polymer chains took a long time to find each other and the response was too slow to be practical. This meant we did not have the sensitivity we needed to determine free Cu(II) in the environment. This study involved a ratiometric signal that was insensitive to factors that affected single intensities. It also demonstrated that polymer conformation-based Cu(II) -sensing solved the problem of Cu(II)-quenching because the Cu(II) and the fluorophore and Cu(II) were far apart.

We found that labeling a single strand of polymer with both a donor and an acceptor led to a change in the extent of FRET when Cu(II) introduced a positive charge into the polymer, causing it to change conformation. However, we also found that the signals from the fluorescent-tagged pNIPAM changed over time because of the untangling of the polymer chain. This ruined the fluorescent measurement. One method we found to solve the problem was to soak the polymer in a ‘good’ solvent (like tetrahydrofuran (THF)) before adding it to water. This accelerated the untangling and made the response more stable.

In this paper, we prepared functionalized pNIPAM using radical addition fragmentation transfer (RAFT) [25]. One advantage of RAFT is that the polymer chain is easy to modify, so we could place the desired functionality on the polymer chain [26,27]. Another is that the chain length of the polymer can be tuned. A third advantage is that RAFT produces polymers with known end groups that can be designed to undergo specific reactions. In this study, we prepared a RAFT agent that included rhodamine as the acceptor. This enabled us to prepare a telechelic polymer with one end attached to a FRET acceptor and the other attached to a FRET donor [28,29]. 

The problem of untangling was addressed by dialyzing the polymer in THF, a good solvent for poly(N-isopropylacrylamide) that accelerates untangling. Poly(N-isopropylacrylamide) was prepared as a backbone and copolymerized with bipyridine, a ligand that strongly binds Cu(II). Cu(II) binding introduces a charge into the polymer backbone [30]. This eventually causes a conformation change in the polymer chain that increases the distance between the donor and acceptor, decreasing the extent of FRET. Our approach retained the advantages of a ratiometric measurement and was not subject to Cu(II)-quenching while giving us a signal that did not change with time. We also found that compared with our previous research, we did not need to elevate the temperature of the LCST of pNIPAM to make it collapse, which was a significant breakthrough. This meant that the whole measurement could be conducted at room temperature.

## 2. Materials and Methods

Azobisisobutyronitrile (AIBN), NIPAM, Rhodamine B, Rhodamine B isothiocyanate, dicyclohexylcarbodiimide (DCC), 4-dimethylaminopyridine (DMAP), 2-cyano-5-hydroxy-2-pentanyl dodecyl carbonotrithioate, fluorescein o-acrylate, 1,3-diaminopropane, and triethylamine (TEA) were purchased from Sigma Aldrich at St. Louis, MO, USA Tris(2-carboxyethyl)phosphine hydrochloride (TCEP) and dioxane were purchased from TCI chemicals at Portland, OR, USA. Acetonitrile, Tetrahydrofuran, and hexane were purchased from J. T. Baker at Phillipsburg, NJ, USA. SpectraPor RC Tubing (cutoff Mw = 3500 Da) was purchased from Repligen at Waltham, MA, USA for dialysis use. N-((4′-Methyl-[2,2′-bipyridin]-4-yl)methyl)-N-propylacrylamide (Bpy) was prepared as previously reported [31,32].

### 2.1. Preparation of the Rhodamine-Tagged Chain Transfer Agent (RCTA)

**Scheme 1:**
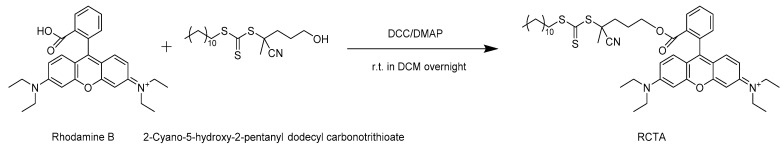
Steglich esterification to prepare the RCTA.

Steglich esterification (shown in Scheme 1) was conducted to obtain a rhodamine-tagged chain transfer agent (RCTA)—(9-(2-(((4-cyano-4-(((dodecylsulfanyl)carbothioyl)sulfanyl)pentyl)oxy)carbonyl)phenyl)-6-(diethylamino)-3H-xanthen-3-ylidene)(diethyl)azanium [33]. Rhodamine B, DCC, DMAP, and 2-cyano-5-hydroxy-2-pentanyl dodecyl carbonotrithioate were added to 10.0 mL of dichloromethane (DCM). After stirring overnight at room temperature, the mixture was purified by a flash column. The desired product was collected and dried via rotary evaporation.

### 2.2. RAFT Polymerization

RAFT polymerization was conducted as described in the literature [25]. To test different factors that could change the ratiometric fluorescent signals, polymers aimed at different chain lengths and different amounts of bipyridine ligands were prepared, as listed in Table 1, where 50mer-8%py and 50mer-6%py means that 8% and 6% of bipyridine monomers were copolymerized within a polymer chain made by 50 NIPAM-repeating units. We maintained the ratio of NIPAM and the bipyridine ligand constant, and changed the amount of the RCTA. In this case, polymers with the same NIPAM/Bpy ratio but with different chain lengths were made. This ensured that the concentration of bipyridine remained the same in the solution for different recipes when comparing the FRET signals. 

NIPAM, RCTA, AIBN, and Bpy (as listed in Table 1) were added into a 100 mL round-bottom flask with 10.0 mL dry dioxane. The whole system was tightly sealed and a freeze-pump–thaw process was conducted 3 times to remove oxygen. After that, the mixture was polymerized at 85 °C for 3 days with constant stirring. The desired polymer was precipitated by adding the solution with a polymer dropwise into 50 mL hexane. This was followed by centrifuging to remove the polymer from the hexane. The resulting polymer was washed several times with hexane, then fully dried under a vacuum for future use.

### 2.3. Preparation of the Telechelic Polymer Indicator

In total, 0.00200 mmol (mass dependent on the polymer chain length) of the polymer derived from the last step was added to 20.0 mL acetonitrile. Next, 0.0300 mmol 1,3-diaminopropane and 0.0200 mmol TCEP were added to the mixture to reduce the trithiocarbonate group to a thiol group [34,35]. After stirring for one day at room temperature, the solution was then dialyzed against 250 mL acetonitrile/water (50 wt.%/50 wt.%) at room temperature. The external solution was changed 2 times per day for 3 days. Then, 0.00200 mmol fluorescein-o-acrylate and 0.0200 mmol TCEP were added to the mixture to tag the thiol end with fluorescein. The mixture was stirred at room temperature overnight and the polymer solution was dialyzed against 250 mL THF [36] at room temperature. The external waste solution was changed 2 times per day for 3 days. In addition to getting rid of the small molecule impurity, this ensured that all the polymers were untangled so the response would not change with time. On the last day of the dialysis, the polymer solution was dialyzed against 250 mL DI water for future use. In total, 5.00 mL of the solution was taken out and lyophilized. After being fully dried, this part of the polymer was weighed to ascertain the concentration in g/L of 5.00 mL of the polymer solution.

### 2.4. Instrumentation

#### 2.4.1. Fluorescent Measurement

Fluorescence was measured using an FS5 spectrofluorometer from Edinburgh Instruments (Edinburgh, UK). Before the experiment, the dialyzed polymer solution was diluted into a 0.0100 g/L solution using 0.100 M of a pH = 6 phosphate buffer. Next, 200 μL of the sample solution and 1800 μL of the solution with the analyte in different concentrations were mixed for the fluorescent test. The excitation wavelength for all the tests was 493 nm. At this excitation wavelength, fluorescein had a maximum emission and we were able to see a rhodamine peak if there was no FRET. The excitation and emission bandwidth of the measurement was 2.00 nm.

#### 2.4.2. Nuclear Magnetic Resonance (NMR) Measurement

The polymerized samples were characterized using a 500 MHz Bruker spectrometer (Billerica, MA, USA). Around 5.00 mg of the samples was added to deuterated chloroform for tests. The NMR data can be seen in the Supporting Information.

## 3. Results and Discussion

**Figure 1:**
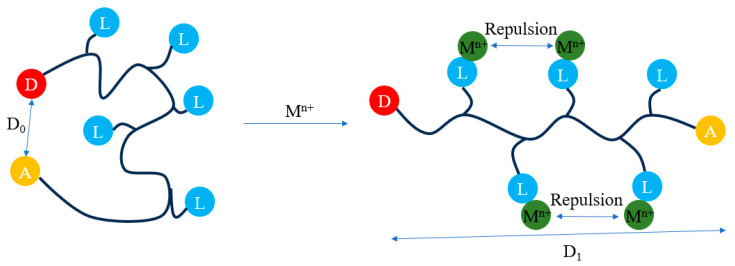
Schematics for copper(II) ion indicator. FRET signal is produced by the conformational change in the polymer. D and A in the picture respectively mean the FRET donor and acceptor; L is the ligand that can bind with the free ions. M^n+^ is the metal ion analytes. Upon metal ions binds with the ligands, the repulsion between the metal ions will change the distance between the FRET pairs.

The structure of the telechelic polymer is shown below. The idea of this design was to use the repulsion of the ions bonded to the polymer to change the distance between the two FRET fluorophores at the two ends of the polymer [37]. Once the copper ions were attached to the copolymerized bipyridine ligands, the polymer chain stretched due to electrostatic repulsion between positive charges. Therefore, the FRET efficiency decreased. The emission intensity from the fluorescent donor increased and the intensity from the acceptor decreased. We measured the ratio of acceptor-to-donor fluorescence. The basic idea is illustrated below in Figure 1.

^1^H-NMR was used to examine the structure of the RCTA and to measure the chain length. The amount of bipyridine was measured (shown in the Supporting Information). By calculating the ratio of the peak at around f_1_ = 7.75 ppm produced by 2 protons on the rhodamine, f_1_ = 4.00 ppm produced by 1 proton on the NIPAM, and f_1_ = 8.60 ppm produced by 2 protons on the bipyridine, we ascertained whether the chain length and the amount of the bipyridine reached our desired value. For example, the ratio of the 50mer-8%py was RCTA:bipyridine-monomer:NIPAM = 1:4.3:52, which was close to our target (1:4:50). Data for the other polymer samples can be seen in Table 2. The NMR spectra can be seen in Figures S2–S7.

### 3.1. Fluorescent Response of FRET-Based Copper Ion Indicator 

**Figure 2:**
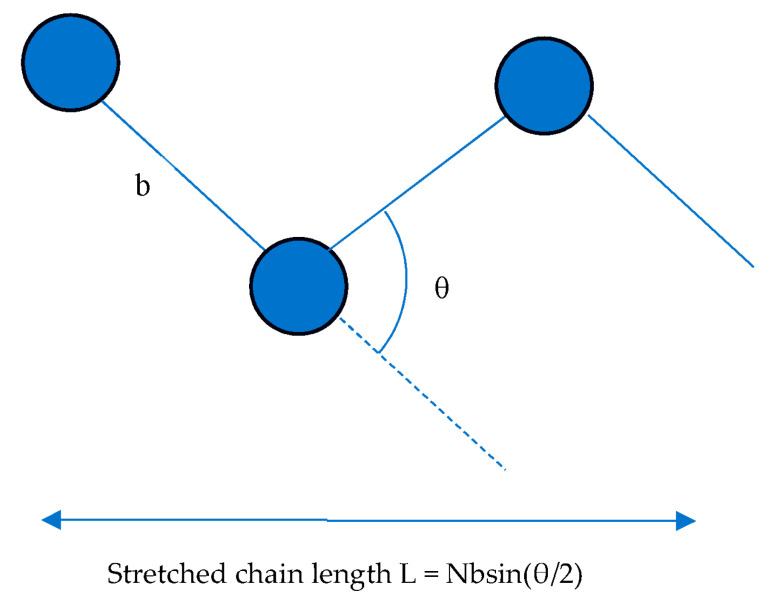
Calculation of stretched chain length.

We used fluorescein (emission wavelength = 512 nm) as the fluorescent donor and rhodamine (excitation wavelength = 540 nm) as the fluorescent acceptor. The Förster critical distance is the distance between an acceptor and a donor when the transfer efficiency is 50%. The Förster critical distance between fluorescein and rhodamine is around 6.60 nm [38]. A molecular dynamic simulation estimated that the gyration radius (R_G_) for a 30mer pNIPAM chain was around 1.00 to 1.20 nm [39], much smaller than the Förster critical distance. That is why we could see a significant acceptor emission peak when we excited the donor. However, when copper(II) binds to a strand, the chain length is stretched. The chain length of a 30mer chain could reach 6.4 nm [40] (Shown at Figure 2, calculated by L = Nbcos(θ/2), where l is the Kuhn length, N is the number of repeating units, b is the bond length, and θ is the bond angle), which was close to the Förster critical distance, causing the emission of the acceptor to diminish. This was the fully stretched length a polymer chain could reach when taking account into the bond length and bond angle [41]. 

### 3.2. FRET Response with Time 

**Figure 3:**
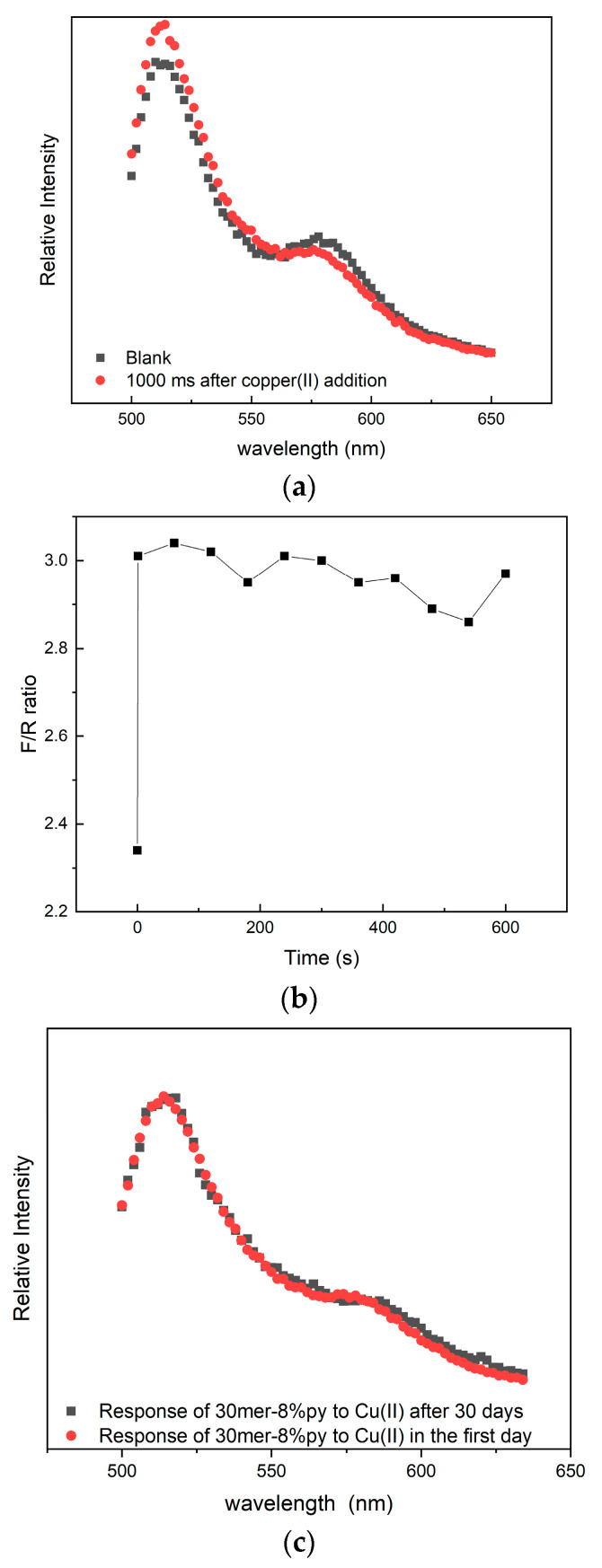
(**a**) The indicator could respond to copper binding within 1000 ms (excitation wavelength = 493 nm). (**b**) F/R ratio versus time in 10 min. There was merely a change after the first second. (**c**) When untangling the moiety of the polymer upon copper binding, the signal was constant even after one month.

The response time of the copper ion indicator was tested by adding 1800 μL of a 10^−5^ M copper(II) pH = 6 phosphate solution into 200 μL of a 0.0100 g/L pH = 6 30mer-8%py solution. The fluorescent response was quick and there was a fluorescent change upon adding the copper ion (shown in Figure 3). The fluorescein/rhodamine peak intensity (F/R ratio) reached a plateau within 1 s. The fluorescent signal after 10 min was tested and was the same as that shown in the graph in Figure S8 This indicated that the response of the polymer sensor was fast and could instantly reach a stable state.

We used THF to solve the untangling issue of pNIPAM that we found in our previous research. pNIPAM untangles slowly in water, which changes the conformation of the polymer in a solution and eventually changes the fluorescence. This process can take two months. A good solvent like THF can untangle pNIPAM in days. We used dialysis against THF before using it as an indicator material. As can be seen in Figure 3c, we added 1800 μL of the 10^−5^ M copper(II) pH = 6 phosphate solution into 200 μL of the 0.0100 g/L pH = 6 30mer-8%py solution, again 30 days after the first measurement. The same signal appeared, which indicated that the response functionality would not change even over one month. This proved that our polymer indicator was not influenced by the change in fluorescence caused by untangling.

### 3.3. Change in the FRET Signal with the Polymer Chain Length

**Figure 4:**
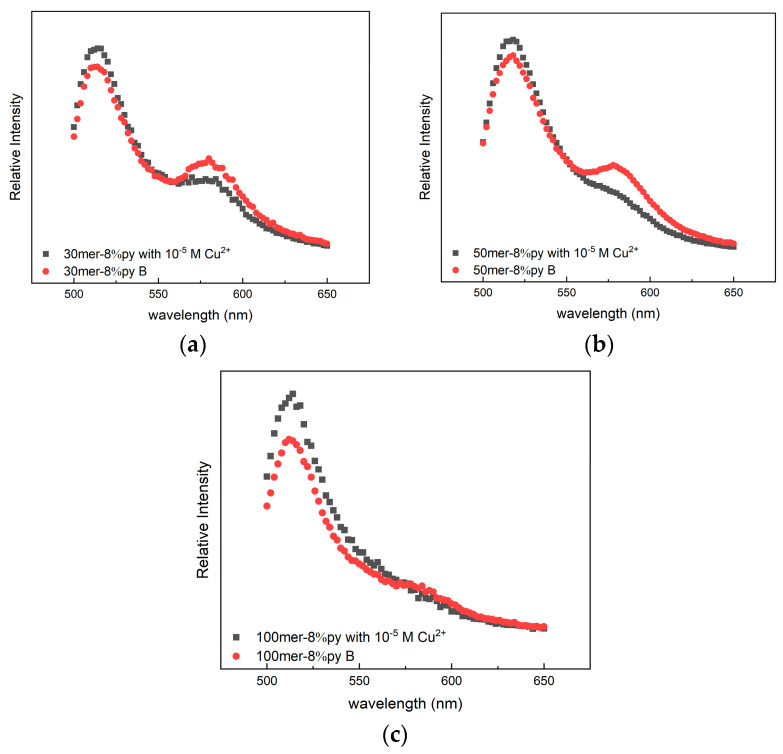
The response change (excitation wavelength = 493 nm) caused by a change in the polymer chain length: (**a**) 30mer-8%py; (**b**) 50mer-8%py; (**c**) 100mer-8%py.

Fluorescent changes caused by the polymer chain length were tested by adding 1800 μL of the 10^−5^ M copper(II) pH = 6 phosphate solution into 200 μL of the 0.0100 g/L pH = 6 30mer-8%py, 50mer-8%py, and 100mer-8%py solutions. As can be seen from the intensity measurement (Figure 4), a huge intensity shift arose in a polymer chain with 30 repeating units and a polymer chain with 50 repeating units. However, if the polymer chain length was enlarged to 100mer, only FRET changes occurred. This indicated that 100mer was too long for a FRET transfer. It agreed with the FRET efficiency curve and that the FRET transfer efficiency would decrease when the distance between the two fluorophores increased [42]. On the other hand, the rhodamine peak disappeared as the polymer lengthened. As the rhodamine was excited by FRET, this was further confirmation that the polymer was performing as we expected.

This could be explained by the persistence length of pNIPAM. The polymer chain was not always fully stretched in the solution. It behaved more like a worm or random coil. In the theory of persistence length, the macromolecule behaves like a flexible elastic rod/beam. If the polymer chain length is larger or lower than its persistence length [43], the polymer chain acts like a rigid rod and if the polymer chain length is around its persistence length, the polymer chain is more like a random coil. The persistence length is half the Kuhn length; in this case, the polymer chain was seen as freely joined. The persistence length of a 30mer pNIPAM was around 3.20 nm, which was smaller than the FRET critical radius of rhodamine and fluorescein. This indicated that it had a very high energy transfer efficiency. However, when the polymer chain reached 50mer, its persistence length was 5.31 nm per meter and 100mer was 10.6 nm, even larger. Therefore, 30mer and 50mer were suitable for the FRET measurement because the FRET signal shifted significantly at this range. We could see that the shift in the 50mer sample was the most significant. However, as the chain length increased, the sensitivity decreased. When the persistence chain length reached 10.6nm, we barely saw any FRET changes.

### 3.4. Change in the FRET Signal with the Amount of Bipyridine on the Chain

**Figure 5:**
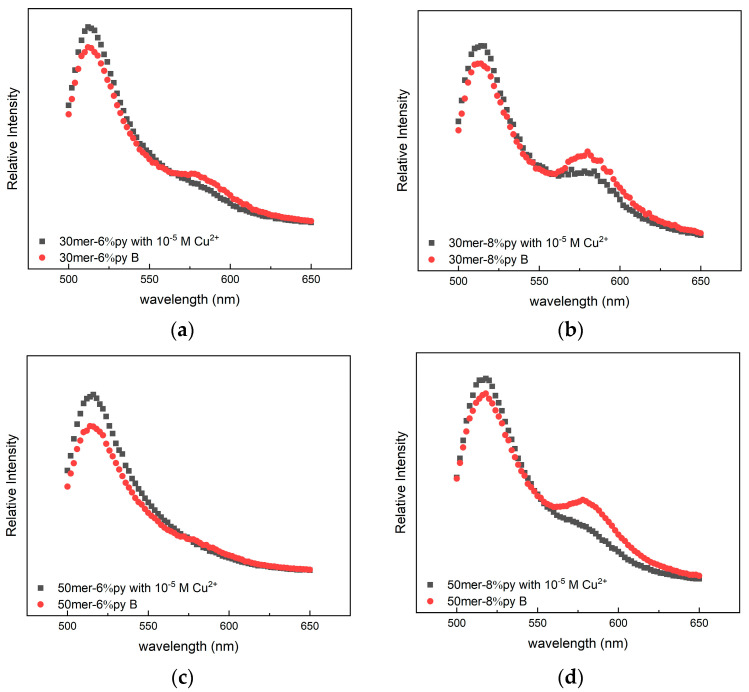
The response change (excitation wavelength = 493 nm) caused by the amount of bipyridine ligand on the polymer. The black line is the polymers with copper ions and the red line is the polymers without copper ions (**a**) 30mer-6%py; (**b**) 30mer-8%py; (**c**) 50mer-6%py; (**d**) 50mer-8%py.

Fluorescent changes caused by different amounts of copper ion binding groups were tested by adding 1800 μL of the 10^−5^ M copper(II) pH = 6 phosphate solution into 200 μL of the 0.0100 g/L pH = 6 samples from Table 1. The comparison of the FRET response for the polymer with different amounts of bipyridine ligands on the chain is presented in Figure 5. As is shown in the figure, if the polymer was equipped with 6% bipyridine ligands, there was hardly any FRET transfer between the two fluorophores. If the bipyridine ligand percentage was increased to 8%, the polymer wrapped the copper ions more tightly. Eventually, this caused a greater FRET shift.

The concept of the electrostatic length was introduced by Odijk [44] and by Skolnick to describe the electrostatic interaction of polyelectrolytes. They showed the interaction between charged monomers on polymer backbones by using the equation below [45]:

$$l_{p}=l_{0}+l_{charge}=l_{0}+\frac{l_{B}f^{2}}{4{\left(\kappa b\right)}^{2}}$$

This equation describes the effects of the charged monomers on the persistence chain length. In the equation, $l_{p}$ is the persistent length affected by the charge in the polymer chain. $l_{0}$ is the original persistent length, which we ascertained in the previous section. b is the bond length. κ is the inverse of the Debye screen length. It describes the screening effect of counterions around the polymer and it is not affected by the chain length and the amount of bipyridine monomers on the chain. f is the fraction of the charged monomer. $l_{B}$ is the Bjerrum length and it describes the repulsion effect caused by the copper ions that are stuck to the polymer. We did not have the method to obtain $l_{B}$ in the equation, but $l_{B}$ is mainly controlled by the relative permittivity and the copper ion concentration in all the sample solutions that were maintained in the same manner. $l_{B}$ did not change too much for these solutions. We can see from the equation that if we change the amount of bipyridine on the chain, the only term that will change is $f^{2}$. When the amount of bipyridine monomer increased from 3 to 4, $f^{2}$ changed from 9 to 16, nearly twice the amount. That is why we observed such a significant change in the signal.

### 3.5. Copper ion FRET Calibration Curve for 30mer-8%py

**Figure 6:**
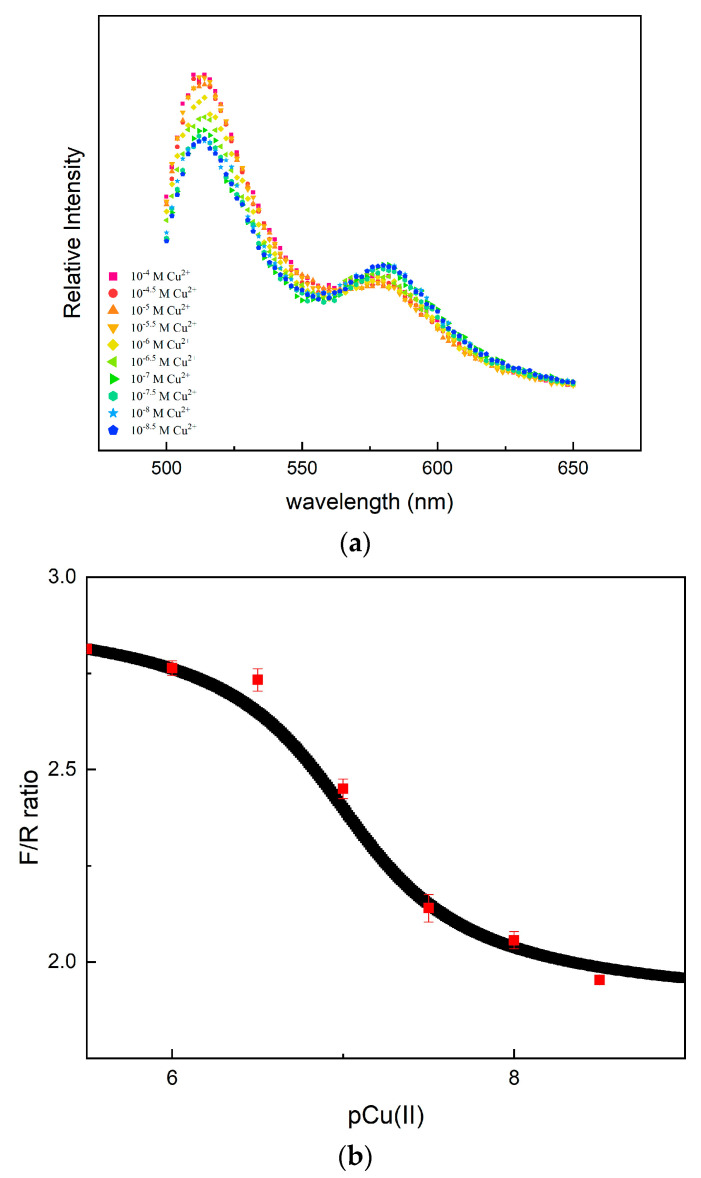

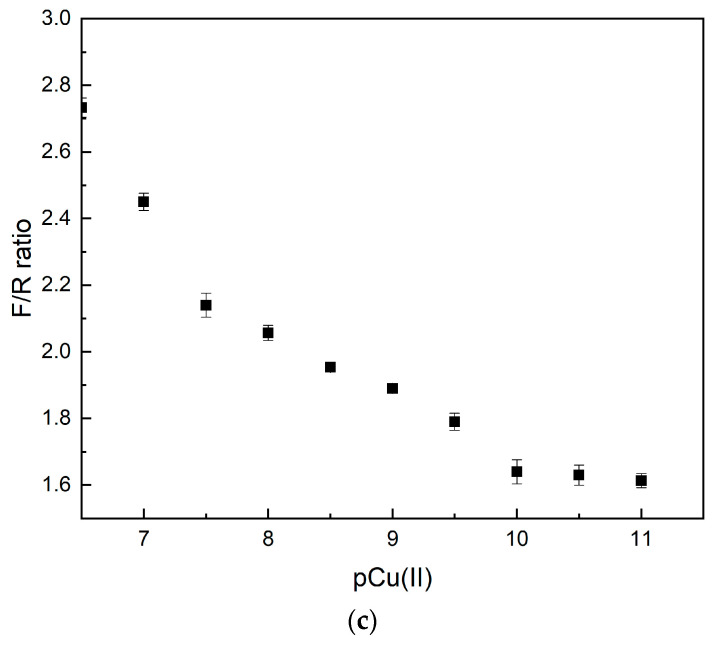
(**a**) Fluorescent response (excitation wavelength = 493 nm) to copper ions in different concentrations. (**b**) Curve for the change in the ratio of fluorescein peak intensity to rhodamine peak intensity (F/R ratio derived from (**a**)) over pCu(II) (−log[Cu^2+^]). The sharpest shift occurred at pCu(II) around 7. (**c**) The F/R ratio at pCu(II) below 7. The F/R ratio reached stability at pCu(II) = 10.

To validate the fluorescence transduction of the copper(II) ion binding, the fluorescence spectra of the indicator were collected using 0.100 M of a pH = 6 phosphate buffer, as shown in Figure 6. Because copper(II) could quench the fluorescence, the sensor could not detect free ions in a much higher value. These data were derived by adding 1800 μL of the pH = 6 phosphate solution with different concentrations of copper(II) (ranging from 10^−4^ M to 10^−11^ M) into 200 μL of the 0.0100 g/L pH = 6 30mer-8%py sample. The error bar was obtained from three repeated tests for the same sample. 

Although the change after adding copper ions was more significant in 50mer-8%py, we used 30mer-8%py for the calibration curve rather than 50mer-8%py because the rhodamine peak at 560 nm for 30mer-8%py was more readable. So, we could obtain a ratio for the peak intensity of these two fluorophores. 

As the concentration of the copper ion decreased, the peak from the FRET donor decreased and the peak from the FRET acceptor increased, as shown in Figure 6a. In this case, the F/R ratio decreased. this indicated that the indicator bonded with more and more free copper ions.

However, the peak intensity for the fluorescent donor and acceptor did not evenly change as the concentration of the copper(II) ions decreased. To determine the effectiveness of this method, we plotted the F/R ratio versus pCu(II) (−log[Cu^2+^]) (Figure 6b). We observed that the change in the F/R ratio versus pCu(II) was not linear. The change was small over 10^−6^ M and below 10^−8^ M. The sharpest shift occurred at pCu(II) around 7. This was the most sensitive range for the sensor. The concentration ratio of the bipyridine monomers and the concentration of copper ions in the equilibrium point in the F/R ratio versus pCu(II) curves was nearly 1:1, which indicated that in this binding ratio, the response of the telechelic polymer indicator had the best performance. Based on the Cu(II)-sensing range that closely corresponded with [Cu(bipy)]^2+^, multiple Bpy ligands did not appear to interact with a single metal ion in the indicators because the Bpy was fixed on the polymer backbone.

When the concentration of the metal ions went beyond 10^−6^ M, the curve gradually reached a plateau. One of the possible reasons was that the concentrated copper ions quenched the fluorescence. Another important reason was that as the concentration of the metal ion increased, it gradually reached the saturation point. This indicated that the polymer could not accept more copper ions, which resulted in the change being less significant. When the concentration of the metal ions went below 10^−9^ M (Figure 6c), the F/R ratio changed slower and slower. The F/R ratio reached stability at pCu(II) = 10. The change in fluorescence at this point was minimal. Below this concentration, the coordination of the copper ions to the metal ion ligand did not lead to a significant conformational change detectable in the two fluorophores at the two ends of the polymer.

### 3.6. Selectivity for other Transition Metals by 50mer-8%py

**Figure 7:**
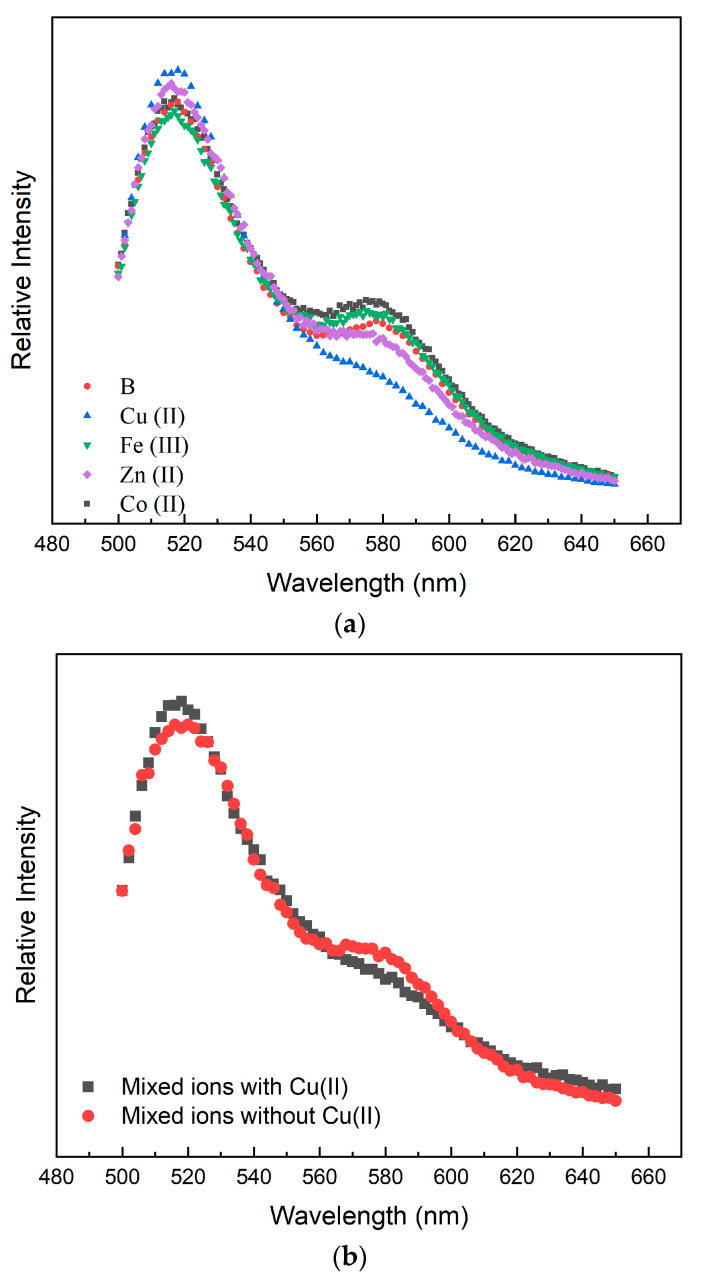
(**a**) Selectivity of the polymer indicator to different metal ions. The copper ion had the largest ratiometric signal change compared with other metal ions. (**b**) Selectivity of copper ions in a solution mixture of Fe^3+^, Zn^2+^, and Co^2+^.

Other metal ions also interact strongly with bipyridine ligands. Therefore, 1800 μL of the pH = 6 phosphate solution with 10^−5^ M of different transition metal ions was added into 200 μL of the 0.0100 g/L pH = 6 50mer-8%py solution for comparison. The ratiometric response of the polymer indicator to various metal ions was evaluated, as shown in Figure 7a. To test the capability of the indicator in a complex ion environment, 1800 μL of the pH = 6 phosphate solution with a mixture of 10^−5^ M Co^2+^, Fe^3+^, and Zn^2+^ was added into 200 μL of the 0.0100 g/L pH = 6 50mer-8%py solution. Cu(II) not only had a relatively large formation constant with Bpy, but also the largest response. The logs of the formation constant for Co(II), Fe(III), and Zn(II) were, separately, 5.8, 4.1, and 5.1 [46], which were lower than that of Cu(II) 8.1. When it appeared on the ratiometric signal change, the copper ion showed the most significant change in comparison with the blank sample (B). So, other metal ions could not interfere with the Cu(II) measurements when at concentrations lower than 10 μM.

Shown in Figure 7b is the selectivity of the sensor when a mixture of different ions was tested to monitor its response in a multi-ion system. Compared with the curve of the ionic solution without copper(II), the indicator had a more significant response in terms of the F/R ratio. However, the ionic mixture interfered with the detection of copper ions. There were three interfering ions, so the total concentration was three times higher than the analyte of interest. The telechelic polymer still responded in this condition, which proved its ability to react against the interference. 

## 4. Conclusions

This free copper ion indicator worked stably at around 10^−7^ M. The lowest concentration that could make a change to the F/R ratio observed with bipyridine as our ligand was ca. 0.1 nanomolar. This concentration level would be adequate when measuring free copper ions. 

Ideally, we wanted the polymer that we used to function as an ‘indicator’. This required that the concentration of free Cu(II) was higher than the indicator concentration, so that the reaction with the indicator was only controlled by the equilibrium constant for the reaction between the indicator and the analyte. In the case of detecting free Cu(II), the indicator concentration had to be 10 picomolar or lower, a very low concentration even for measurements using fluorescence. Note also that by ‘indicator’ concentration we mean the concentration of the ligand. In the telechelic polymer that we used, we had three or four bipyridines per one set of fluorophores, so the fluorophore concentration was a factor of three or four lower than the ‘indicator’ concentration. 

The other extreme occurred if we used a very high-affinity ligand in our polymer. In this case, the observed signal only depended on stoichiometry, i.e., the fraction of ligands that were attached to Cu(II). This was easier to experimentally realize because the ligand concentration had to be similar to the analyte concentration. The problem with this approach was that we perturbed the equilibria under study. A strong ligand pulls Cu(II) away from weaker ligands and we could not be sure that we were actually measuring free Cu(II). 

We believed that the final solution involved an intermediate-strength ligand at a relatively high concentration, or 0.10 nanomolar. This concentration was too high for the indicator to function as a true indicator. Instead, the response to free Cu(II) had to be determined by calibration. 

## Figures and Tables

**Table 1:** Recipe for the telechelic polymer indicator.

	RCTA	NIPAM	AIBN	Bpy
100mer-8%py	5.12 mg (0.00602 mmol)	140 mg (1.24 mmol)	0.102 mg (0.000621 mmol)	30.0 mg (0.102 mmol)
100mer-6%py	5.12 mg (0.00602 mmol)	140 mg (1.24 mmol)	0.102 mg (0.000621 mmol)	22.5 mg (0.0773 mmol)
50mer-8%py	6.14 mg (0.00721 mmol)	140 mg (1.24 mmol)	0.122 mg (0.000682 mmol)	30.0 mg (0.102 mmol)
50mer-6%py	6.14 mg (0.00721 mmol)	140 mg (1.24 mmol)	0.122 mg (0.000682 mmol)	22.5 mg (0.0773 mmol)
30mer-8%py	10.2 mg (0.0119 mmol)	140 mg (1.24 mmol)	0.203 mg (0.00124 mmol)	30.0 mg (0.102 mmol)
30mer-6%py	10.2 mg (0.0119 mmol)	140 mg (1.24 mmol)	0.203 mg (0.00124 mmol)	22.5 mg (0.0773 mmol)

**Table 2:** Composition determined by ^1^H-NMR.

	Target Composition (RCTA:Bipyridine-Ligand:NIPAM)	Actual Composition (RCTA:Bipyridine-Ligand:NIPAM
100mer-8%py	1:8:100	1:7.0:117
100mer-6%py	1:6:100	1:6.0:105
50mer-8%py	1:4:50	1:4.3:64
50mer-6%py	1:3:50	1:2.5:52
30mer-8%py	1:2.4:30	1:2.4:25
30mer-6%py	1:1.8:30	1:1.6:26

## Data Availability

Data is contained within the article or Supplementary Material.

## Supplementary Materials

The following supporting information can be downloaded at: https://www.mdpi.com/article/10.3390/s23239476/s1. Figure S1. ^1^H-NMR for RCTA (the green lines are the integration of the peaks. The integrated values are shown in the bottom of the figure. They were used to compare different functional groups on the polymer chain). Figure S2. ^1^H-NMR for 30mer-4py; the integrated value for a:b:c was around 1:3.3:52. Because 2 H in the molecule formed a, 1 formed b, and 2 formed c, the RCTA:bipyridine-monomer:NIPAM ratio = 1:1.6:26 (target was 1:1.8:30). Figure S3. ^1^H-NMR for 30mer-3py; the integrated value for a:b:c was around 1:4.7:50. Because 2 H in the molecule formed a, 1 formed b, and 2 formed c, the RCTA:bipyridine-monomer:NIPAM ratio = 1:2.4:25 (target was 1:2.4:30). Figure S4. ^1^H-NMR for 50mer-4py; the integrated value for a:b:c = 1:8.5:105. Because 2 H in the molecule formed a, 1 formed b, and 2 formed c, the RCTA:bipyridine-monomer:NIPAM ratio = 1:4.3:52 (target was 1:4:50). Figure S5. ^1^H-NMR for 50mer-3py; the integrated value for a:b:c was around 1:5:128. Because 2 H in the molecule formed a, 1 formed b, and 2 formed c, the RCTA:bipyridine-monomer:NIPAM ratio = 1:2.5:64 (target was 1:3:50). Figure S6. ^1^H-NMR for 100mer-4py; the integrated value for a:b:c was around 1:14:234. Because 2 H in the molecule formed a, 1 formed b, and 2 formed c, the RCTA:bipyridine-monomer:NIPAM ratio = 1:7:117 (target was 1:8:100). Figure S7. ^1^H-NMR for 100mer-3py; the integrated value for a:b:c was around 1:12:209. Because 2 H in the molecule formed a, 1 formed b, and 2 formed c, the RCTA:bipyridine-monomer:NIPAM ratio = 1:6:105 (target was 1:6:100). Figure S8. 1000 ms and 10 min after copper(II) binding on the telechelic polymer.
