# Supplementary material for: A Telechelic Fluorescent Indicator Based on Polymer Conformational Change for Free Copper(II) Ions
Source: Sensors (Basel). 2023 Nov 28;23(23):9476. doi: 10.3390/s23239476 (PMC10708570; doi:10.3390/s23239476)
Supplement: Supplementary file 1 [file sensors-23-09476-s001.zip › sensors-2712180-supplementary.pdf]

A telechelic fluorescent indicator based on polymer conformational change for free copper(II) ions

Yuan Chen, Bo Si, Noah Cote, Roy Planalp, W. Rudolf Seitz*

Department of Chemistry, University of New Hampshire, Durham, New Hampshire 03824

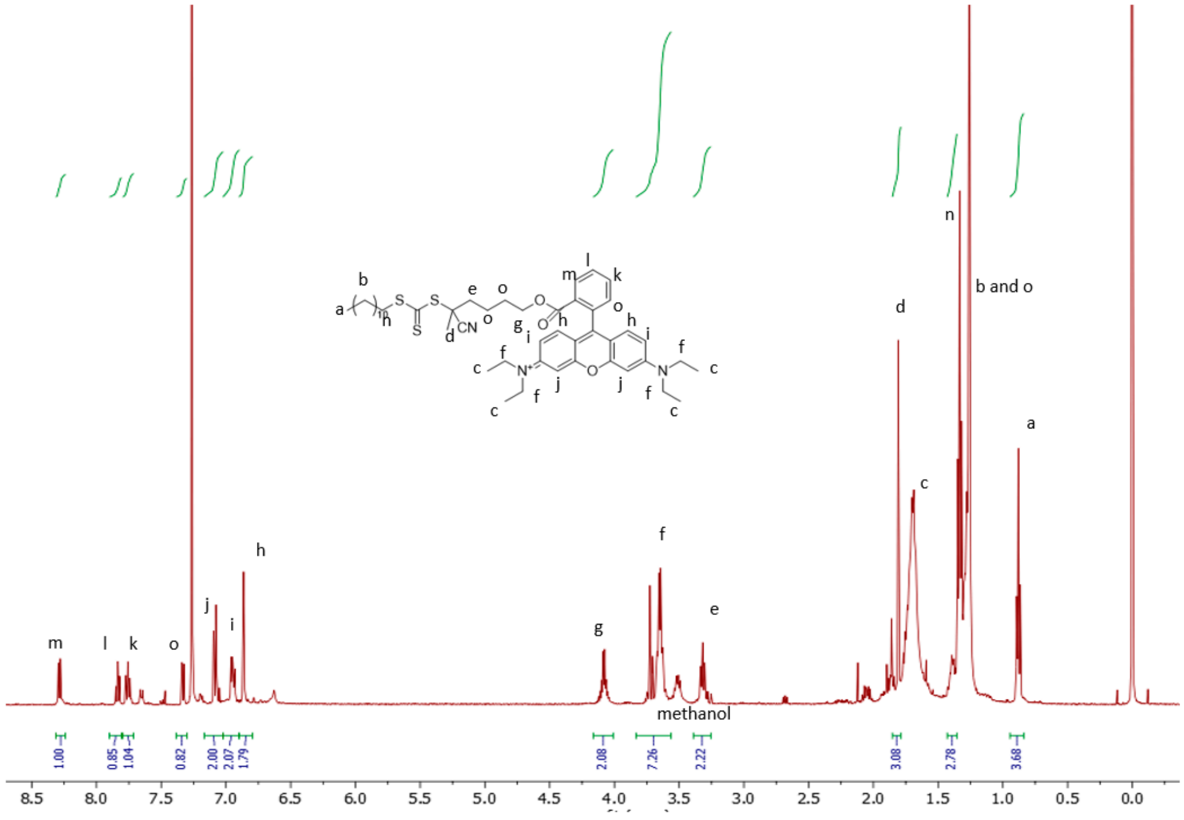

Figure S1. ^{1}H-NMR for RCTA (The green lines are the integration of the peaks. The integrated values are shown in the bottom of the figure. They are used to compare different functional groups on the polymer chain.)

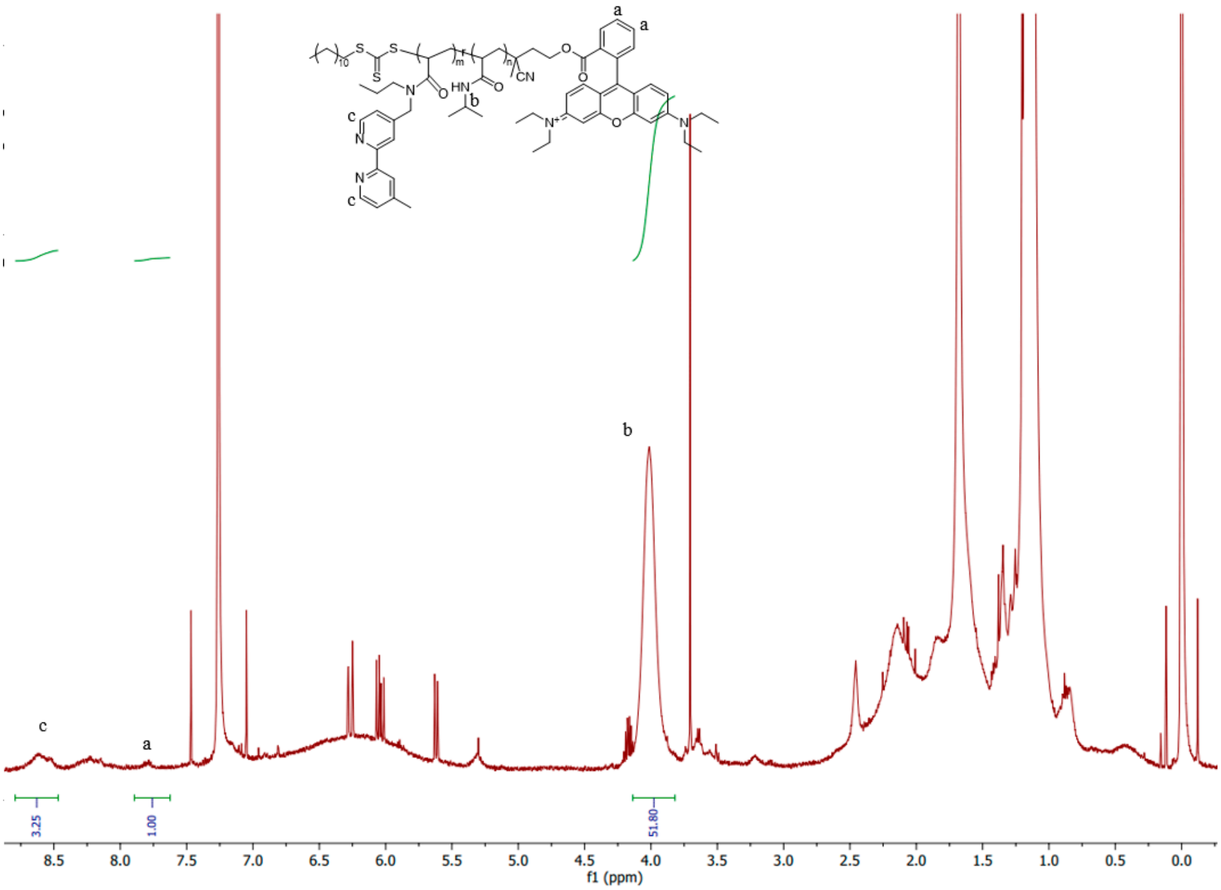

Figure S2. ^{1}H-NMR for 30mer-4py, the integrated value for a:b:c is around 1:3.3:52. Because 2 H in the molecule forms a, 1 forms b, and 2 forms c, the RCTA: bipyridine monomer: NIPAM=1:1.6:26 (target at 1:1.8:30)

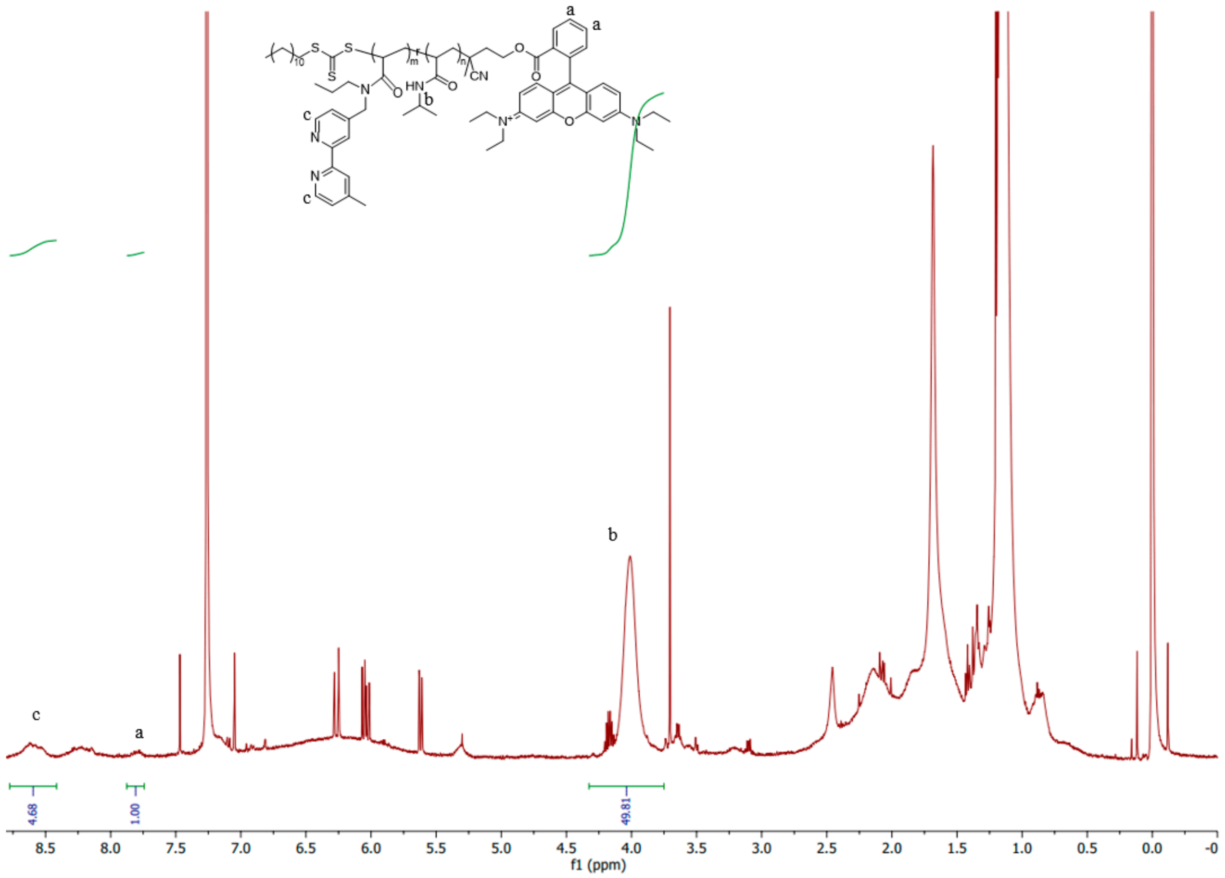

Figure S3. ^{1}H-NMR for 30mer-3py, the integrated value for a:b:c is around 1:4.7:50. Because 2 H in the molecule forms a, 1 forms b, and 2 forms c, the RCTA: bipyridine monomer: NIPAM=1:2.4:25 (target at 1:2.4:30)

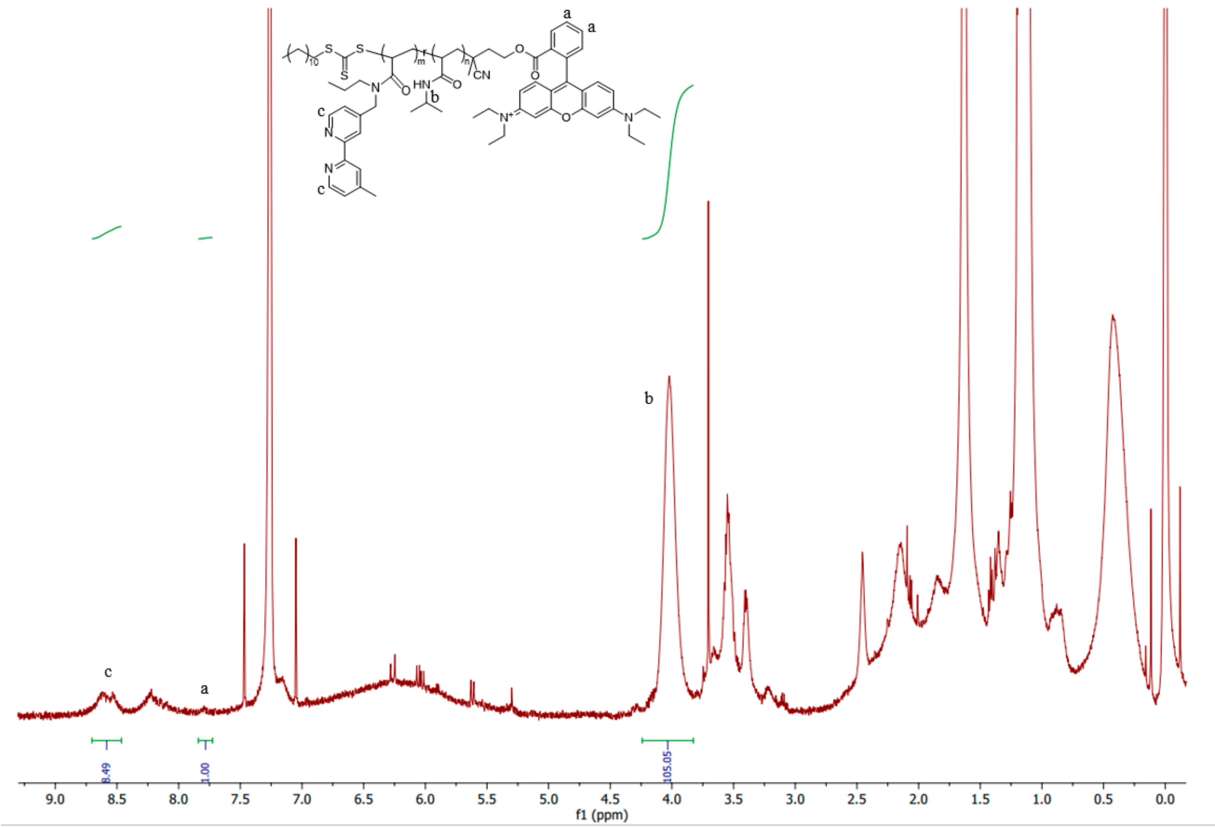

Figure S4. ^{1}H-NMR for 50mer-4py, the integrated value for a:b:c=1:8.5:105. Because 2 H in the molecule forms a, 1 forms b, and 2 forms c, the RCTA: bipyridine monomer: NIPAM=1:4.3:52 (target at 1:4:50)

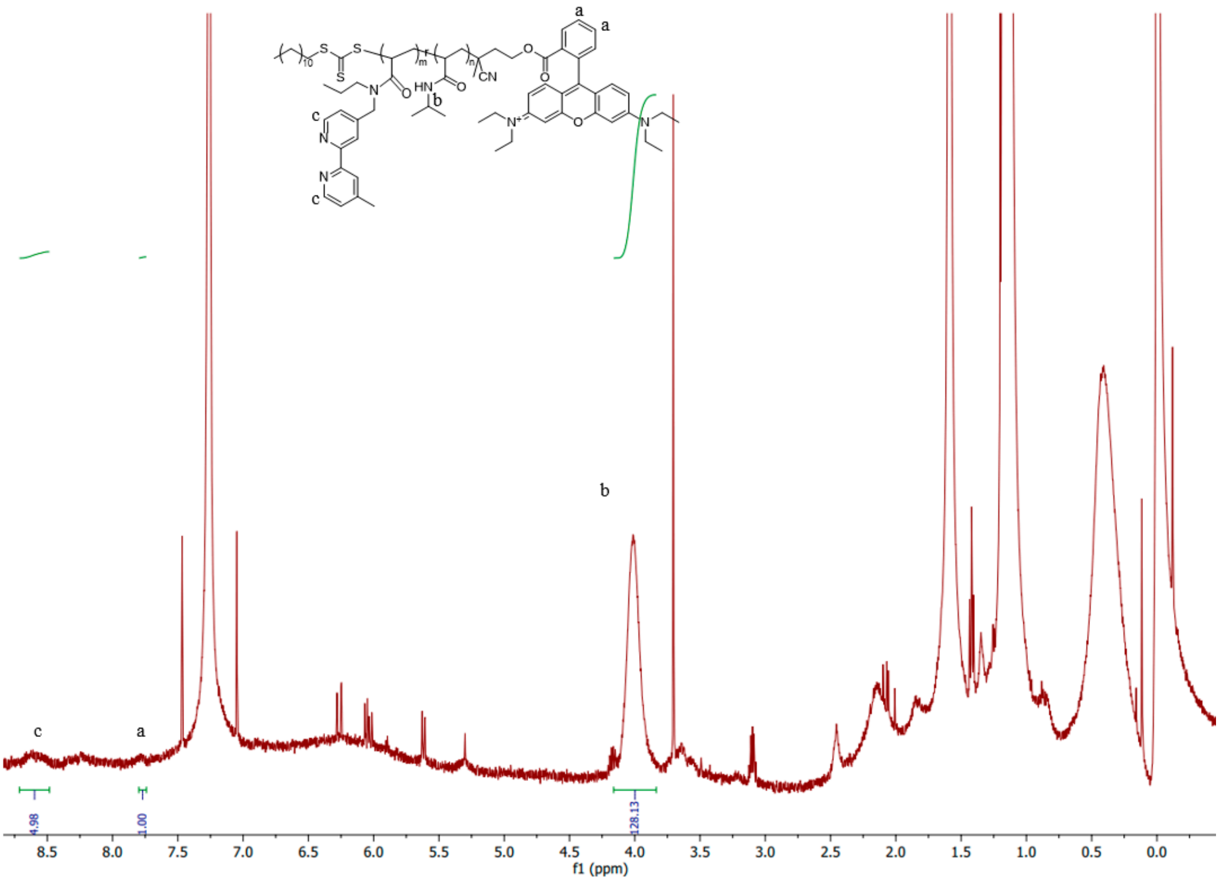

Figure S5. ^{1}H-NMR for 50mer-3py, the integrated value for a:b:c is around 1:5:128. Because 2 H in the molecule forms a, 1 forms b, and 2 forms c, the RCTA: bipyridine monomer: NIPAM= 1:2.5:64 (target at 1:3:50)

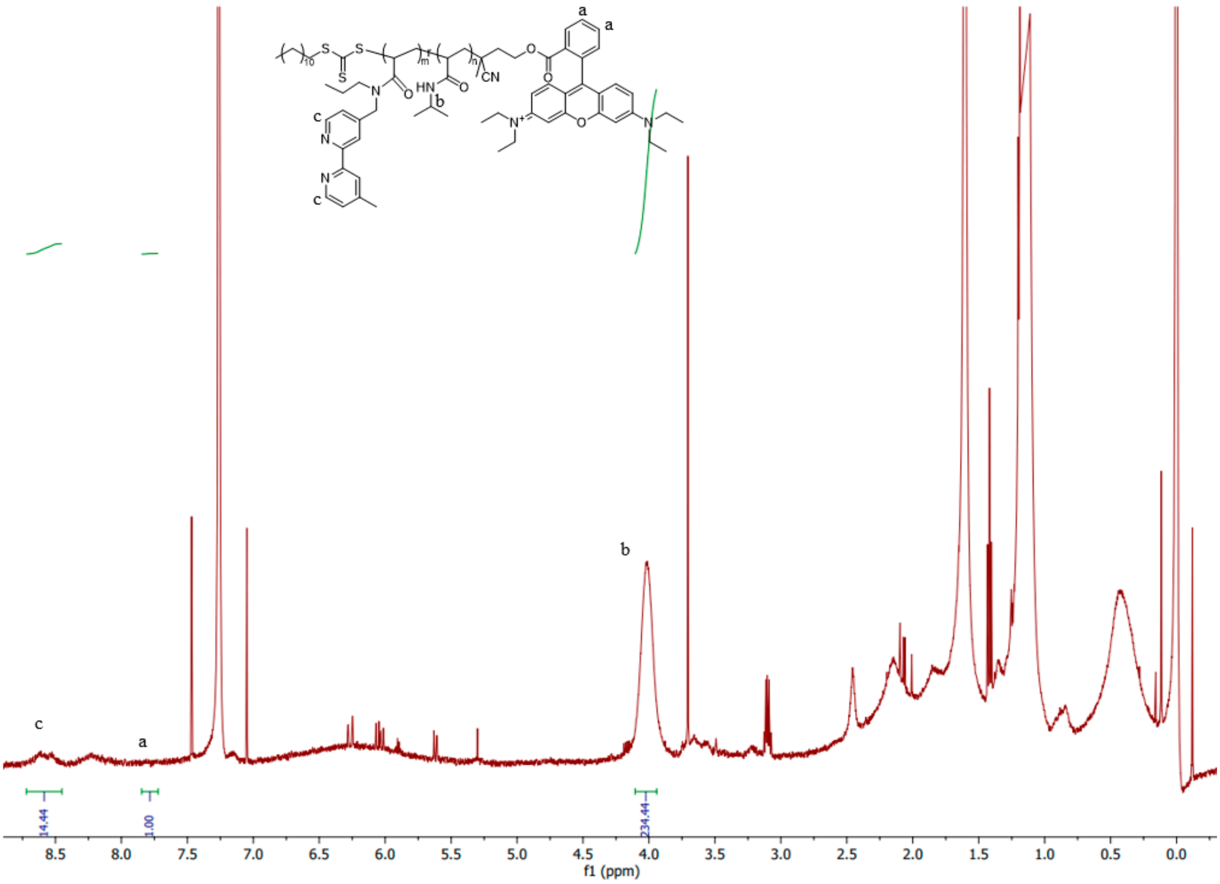

Figure S6. ^{1}H-NMR for 100mer-4py, the integrated value for a:b:c is around 1:14:234. Because 2 H in the molecule forms a, 1 forms b, and 2 forms c, the RCTA: bipyridine monomer: NIPAM=1:7:117 (target at 1:8:100)

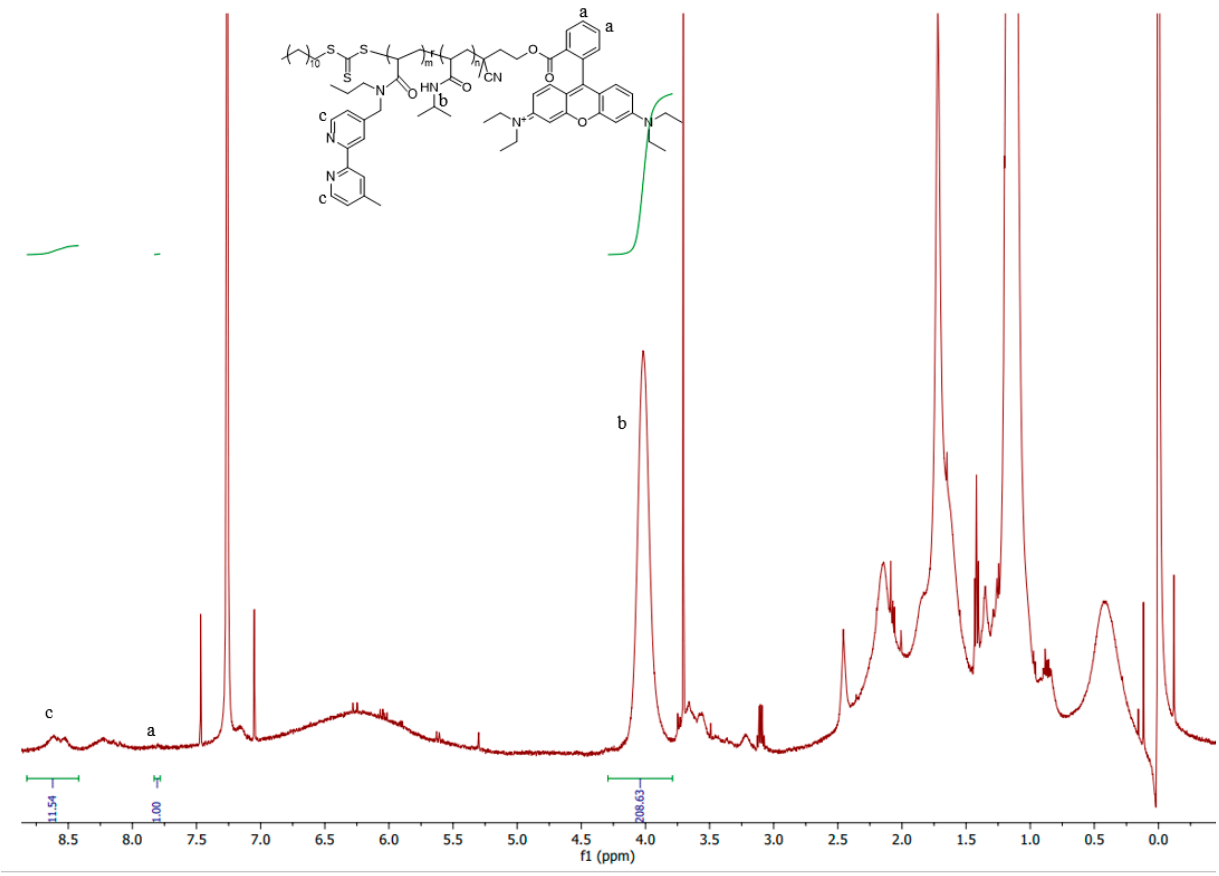

Figure S7. ^{1}H-NMR for 100mer-3py, the integrated value for a:b:c is around 1:12:209. Because 2 H in the molecule forms a, 1 forms b, and 2 forms c, the RCTA: bipyridine monomer: NIPAM=1:6:105 (target at 1:6:100)

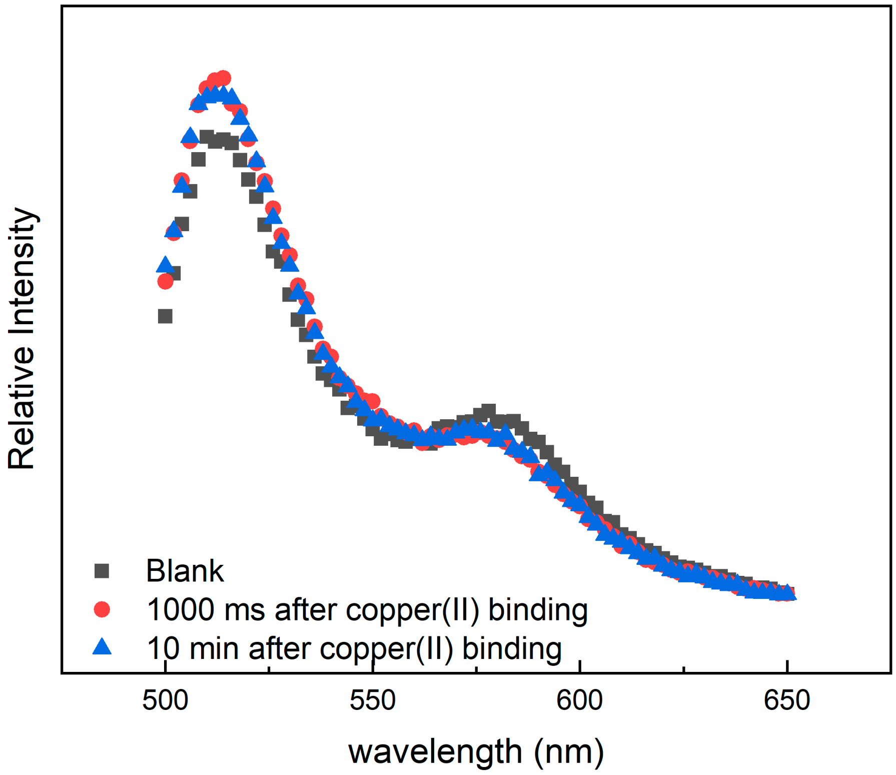

Figure S8. 1000ms and 10 min after copper (II) binding on the telechelic polymer
